# Voxel- and surface-based morphometry in the cortical thickness and cortical and subcortical gray matter volume in patients with mild-to-moderate Alzheimer’s disease

**DOI:** 10.3389/fnagi.2025.1546977

**Published:** 2025-06-25

**Authors:** Kaidi Li, Dingling Xie, Zhengyong Zhang, Chunyu Fu, Chunyang Li

**Affiliations:** ^1^Department of Neurology, Affiliated Hospital of Inner Mongolia Medical University, Hohhot, China; ^2^Inner Mongolia Regional Center for Neurological Disorders, Hohhot, China; ^3^Department of Neurology, Xianning Central Hospital (The First Affiliated Hospital of Hubei University of Science and Technology), Xianning, China; ^4^School of Public Health, Xinxiang Medical College, Xinxiang, China; ^5^Department of Neurosurgery, Affiliated Hospital of Inner Mongolia Medical University, Hohhot, China; ^6^Department of Neurology, Inner Mongolia Brain Hospital (Third Hospital), Hohhot, China

**Keywords:** SBM, VBM, grey matter volume (gmv), cortical thickness (CT), Alzheimer’s disease

## Abstract

**Aim:**

This study aimed to investigate alterations in whole-brain cortical thickness (CT) and cortical and subcortical gray matter volume (GMV) in patients with Alzheimer’s disease (AD) compared with healthy controls (HC) using voxel-based morphometry (VBM) and surface-based morphometry (SBM). Furthermore, we sought to develop a combined predictive model based on these neuroimaging markers and assess their potential clinical utility for the early detection and diagnosis of AD.

**Methods:**

A total of 42 patients diagnosed with mild-to-moderate AD and 49 demographically matched HC were recruited for this study. VBM and SBM analyses were performed on three-dimensional T1-weighted magnetization-prepared rapid gradient echo (3D T1-MPRAGE) imaging sequences to identify brain regions that exhibited statistically significant differences between the AD and HC groups. Brain regions showing significant group differences were selected as the regions of interest (ROIs). Pearson’s correlation analysis was used to assess the relationship between extracted neuroimaging metrics (CT, cortical GMV, and subcortical GMV) and cognitive performance. Predictive models were constructed using CT (from SBM), cortical GMV, and subcortical GMV (from VBM) metrics derived from ROIs, both individually and in combination. Model performance in discriminating between patients with AD and HCs was evaluated using a receiver operating characteristic (ROC) curve analysis.

**Results:**

Compared to HCs, patients with AD exhibited significant CT reductions primarily in the transverse temporal gyrus, superior temporal gyrus, supramarginal gyrus, insula, temporal pole, entorhinal cortex, and fusiform gyrus. Significant GMV reductions in patients with AD were observed predominantly in the hippocampus, parahippocampal gyrus, posterior temporal lobe, inferior temporal gyrus, middle temporal gyrus, limbic lobe structures, fusiform gyrus, amygdala, and thalamus, as detected by VBM analysis. Extracted CT, cortical GMV, and subcortical GMV measurements from the ROIs demonstrated significant positive correlations with both MMSE and MoCA scores.

**Conclusion:**

In patients with AD, VBM and SBM showed overlapping cortical GMV and CT reductions. Volume/thickness reduction was correlated with lower MMSE/MoCA scores, confirming functional relevance. ROC analysis revealed that combining CT and GMV improved cognitive impairment prediction compared to single measures. This integrated approach may enhance clinical diagnosis and early risk identification of AD.

## Introduction

Alzheimer’s disease (AD) is a progressive neurodegenerative disorder characterized by central nervous system degeneration, with brain atrophy (BA) being a prominent neuroimaging hallmark of brain atrophy. Extensive research indicates that BA represents a continuous and progressive process (Braak and Braak, 1991; Knudsen et al., 2022). Pathological changes associated with BA typically originate in the hippocampus and entorhinal cortex (Devanand et al., 2012; Sakurai et al., 2019; Huang et al., 2020), subsequently spreading to the temporal and parietal lobes and ultimately encompassing the entire brain (Chrzan et al., 2019; Sun et al., 2019; Contador et al., 2021; Zhang et al., 2021).

Current structural neuroimaging methods for quantifying BA include surface-based morphometry (SBM) and voxel-based morphometry (VBM). Numerous studies have demonstrated that both SBM and VBM are effective for identifying brain regions affected by atrophy (Knudsen et al., 2022; Zhuang et al., 2022; Longhurst et al., 2023). However, a majority of these investigations have employed a single analytical approach, typically assessing either cortical thickness (CT) derived from SBM or gray matter volume (GMV) derived from VBM, rather than integrating metrics from both methodologies. This distinction is significant because CT measures the distance between the pial surface and white matter boundary, reflecting cortical gray matter layer thickness, whereas GMV quantifies the total volume of gray matter within a specific region.

Recent studies have reported structural, functional, and molecular alterations in the brains of patients with dementia with either gradual progression or different temporal alteration trajectories of each multiparametric metric that depend on the disease stage, severity, and quantification accuracy (Zhou, 2021). In AD, CT is typically reduced in the temporal and parietal regions compared to healthy controls (Yuan et al., 2025). Similarly, GMV is also diminished in these areas, particularly within the medial temporal lobe, where key limbic structures such as the parahippocampal regions are notably affected (Ramirez et al., 2025). Furthermore, cortical atrophy is evident in specific structures, including the inferior parietal lobule, middle temporal gyrus, precuneus, and insula in individuals with AD (Kim et al., 2025). Collectively, these structural alterations are crucial for elucidating the progression of AD and their impact on brain morphology and function.

The integration of SBM and VBM enables a more comprehensive assessment of changes in brain volume and cortical thickness, thereby enhancing the accuracy of early diagnosis (Yan et al., 2024). Moreover, these techniques can be employed to identify specific regional brain features associated with cognitive decline, which aids in differentiating between distinct stages of AD, such as Mild Cognitive Impairment (MCI) and early AD (Chen et al., 2024). Consequently, the combined application of SBM and VBM shows potential as a diagnostic biomarker for predicting AD progression, thereby providing a basis for timely intervention and treatment strategies (Cui et al., 2024).

To address the limitations of single-modality analysis, the present study combined the SBM and VBM approaches to comprehensively analyze alterations in CT, cortical GMV, and subcortical GMV in patients diagnosed with mild-to-moderate AD, comparing them to cognitively normal elderly controls matched for age and educational level. Brain regions exhibiting significant atrophy in the AD group compared with the controls were defined as regions of interest (ROIs). Subsequently, we investigated the association between these neuroanatomical measures within the identified ROIs and cognitive performance, as assessed using the Mini-Mental State Examination (MMSE) and Montreal Cognitive Assessment (MoCA). Finally, we developed classification models utilizing CT, cortical GMV, and subcortical GMV measures—both individually and in combination—to differentiate patients with AD from healthy controls and evaluated the diagnostic performance of these models using receiver operating characteristic (ROC) curve analysis.

## Methods

### Study population

The study participants were recruited from the Department of Neurology at the Affiliated Hospital of Inner Mongolia Medical University and the Department of Neurology at the Central Hospital of Xianning City, Hubei Province, between August 2022 and June 2024. A total of 42 patients diagnosed with mild-to-moderate Alzheimer’s disease (AD) constituted the AD group. Concurrently, 49 cognitively normal individuals were recruited as healthy controls (HC).

### Inclusion and exclusion criteria

#### AD group

The diagnosis of AD was established based on the criteria published by the National Institute on Aging and the Alzheimer’s Association (NIA-AA) in 2011 (Jack et al., 2011) and the International Working Group (IWG) IWG-2 study criteria from 2014 (Dubois et al., 2014), additionally referencing the Diagnostic and Statistical Manual of Mental Disorders, Fourth Edition, Text Revision (DSM-IV-TR) criteria (Cooper, 2001).

##### Inclusion criteria

(1) Mini-Mental State Examination (MMSE) score between 15 and 25 (inclusive), (2) Montreal Cognitive Assessment (MoCA) score < 22 (Roalf et al., 2013), (3) Clinical Dementia Rating Sum of Boxes (CDR-SB) score ≥ 1, (4) Alzheimer’s Disease Cooperative Study - Activities of Daily Living (ADCS-ADL) score ≥ 22, (5) Absence of significant neurological or psychiatric comorbidities other than AD, and (6) Medial Temporal lobe Atrophy (MTA) scale score ≥ 1.5 (utilizing age-adjusted values: ≥1.0 for ages 55–64, ≥1.5 for ages 65–74, ≥2.0 for ages 75–84).

##### Exclusion criteria

(1) Contraindications to magnetic resonance imaging (MRI); (2) Inability to cooperate with necessary examinations, including laboratory tests and neuropsychological assessments; and (3) Presence of other conditions potentially causing cognitive impairment, such as vascular dementia, frontotemporal dementia, Lewy body dementia, or other dementia types; severe anemia, thyroid dysfunction, folic acid, vitamin B12 deficiency, or other relevant metabolic diseases; major psychiatric disorders; or cognitive impairment secondary to poisoning, trauma, infection, or medication.

#### HC group

Healthy controls (HC) comprised cognitively normal elderly individuals aged 50–85 years, selected to match the AD group in terms of general demographic characteristics.

##### Inclusion criteria

(1) Normal findings on structural brain MRI, (2) No subjective or objective evidence of memory loss or other cognitive decline (MMSE score ≥ 26, MoCA score ≥ 26), (3) Absence of significant metabolic disorders relevant to cognitive function, and (4) No history of major psychiatric or neurological diseases.

##### Exclusion criteria

Same as those applied to the AD group.

### Clinical data collection

#### General clinical data

Written informed consent was obtained from all participants or their legal guardians prior to enrollment. Demographic and clinical information, including age, sex, handedness, education level, history of memory loss, medical history, family history, and neurological examination results, was collected for each participant. The study protocol was reviewed and approved by the Ethics Committee of the Affiliated Hospital of Inner Mongolia Medical University (Approval Number: KY2024065).

#### Laboratory examinations

Fasting venous blood samples were collected from all the participants for a comprehensive panel of laboratory tests. These included complete blood count, coagulation profile, serum electrolytes, liver and kidney function tests, myocardial enzyme spectrum, thyroid function tests, blood glucose, lipid profile, tumor markers, and screening for hepatitis, syphilis, and HIV. These examinations aim to exclude secondary causes of cognitive impairment, including infectious, metabolic, neoplastic, and autoimmune conditions.

#### MRI acquisition

Brain MRI was performed using a 3.0 Tesla Siemens Magnetom Verio scanner. High-resolution structural images were acquired using a three-dimensional magnetization-prepared rapid gradient-echo (3D-MPRAGE) T1-weighted imaging (T1WI) sequence covering the whole brain (from the cranial apex to the foramen magnum). The acquisition parameters were as follows: Repetition Time (TR) = 2,530 ms; Echo Time (TE) = 2.22 ms; flip angle (FA) = 7°; Field of View (FOV) = 224 mm x 224 mm; matrix size = 224 × 224; Voxel Size (VS) = 1 mm x 1 mm x 1 mm; number of slices = 176; slice thickness = 0.9 mm; slice gap = 1.9 mm.

### Image processing

#### Surface-based morphometry (SBM) and voxel-based morphometry (VBM)

Image processing was conducted using Statistical Parametric Mapping software (SPM12, v7771; The Wellcome Centre for Human Neuroimaging, London, UK) and the Computational Anatomy Toolbox (CAT; 12.7-Beta, r1615; Structural Brain Mapping Group, Jena University Hospital, Germany) implemented in Matrix Laboratory (MATLAB) (R2018 version; The MathWorks, Inc., Natick, MA, USA). Digital Imaging and Communications in Medicine (DICOM) images were converted to the NIFTI format. Images were manually reoriented, setting the anterior commissure as the origin for standardization.

#### SBM analysis (cortical thickness)

Cortical thickness (CT) was estimated using the projection-based thickness method implemented in CAT12, which reconstructs the central cortical surface for each hemisphere. The surface data were subsequently resampled and smoothed using a Gaussian kernel with a full width at half maximum (FWHM) of 15 mm. Extracted CT values were compared between the two study groups using a general linear model (GLM) framework, specifically employing independent two-sample t-tests vertex-wise across the cortical surface. Statistical inference was performed using Threshold-Free Cluster Enhancement (TFCE) to identify significant clusters (Salimi-Khorshidi et al., 2011), potentially employing an initial cluster-forming threshold of *p* < 0.001 (uncorrected). The final statistical threshold was set at *p* < 0.05, corrected for multiple comparisons across the surface using the Family-Wise Error (FWE) rate at the cluster level. This analysis yielded statistical maps highlighting the regions of significant between-group differences in CT.

#### VBM analysis (gray matter volume)

Participants’ 3D-T1WI images were segmented into gray matter (GM), white matter (WM), and cerebrospinal fluid (CSF) probability maps using the CAT12 toolbox. The GM maps were spatially normalized to the Montreal Neurological Institute (MNI) space using the ICBM152 template via the Diffeomorphic Anatomical Registration Through Exponentiated Lie algebra (DARTEL) algorithm and modulated to preserve volumetric information. Normalized, modulated GM maps were resampled to an isotropic voxel size of 1.5 × 1.5 × 1.5 mm^3^ and smoothed using an 8 mm FWHM Gaussian kernel. Between-group comparisons of the regional Gray Matter Volume (GMV) were conducted using a GLM framework with independent two-sample t-tests. The Total Intracranial Volume (TIV) calculated for each participant was included as a covariate in the statistical model to account for variations in head size. Statistical significance was assessed using TFCE with a threshold of *p* < 0.05 (uncorrected). FWE-corrected at cluster level (*p* < 0.05, uncorrected). This analysis produced statistical maps indicating regions with significant between-group differences in GMV.

### Statistical analysis

Statistical analyses of the demographic, clinical, and ROI-based data were performed using Statistical Package for the Social Sciences (SPSS) version 29.0 (IBM Corp., Armonk, NY, USA). Continuous variables were compared between the AD and HC groups using one-way analysis of variance (ANOVA) and are presented as mean ± standard deviation (SD). Categorical variables (e.g., sex and handedness) were compared using the chi-squared (χ^2^) test or Fisher’s exact test. A *p*-value < 0.05 was considered statistically significant for these comparisons.

Following whole-brain SBM and VBM analyses, brain regions demonstrating statistically significant differences between groups (in CT or GMV) were defined as regions of interest (ROIs). Mean CT values (from SBM-defined ROIs) and mean GMV values (from VBM-defined ROIs separated into cortical and subcortical sources) were extracted for each participant from the respective ROIs.

Pearson’s correlation analyses were conducted to investigate the associations between the extracted neuroimaging metrics (mean CT and GMV within the ROIs) and cognitive performance scores (MMSE and MoCA) across all participants.

Finally, receiver operating characteristic (ROC) curve analyses were performed to assess the diagnostic utility of the mean ROI-based CT and GMV values in distinguishing patients with AD from HCs. The area under the curve (AUC) was calculated for each metric. Additionally, a combined model incorporating multiple neuroimaging metrics was evaluated using ROC analysis to determine whether the combination of markers improved the diagnostic performance.

## Results

### Participant characteristics and neuropsychological performance

A total of 91 participants were enrolled in the study; their demographic and clinical characteristics are summarized in Table 1. The AD and HC groups were comparable in terms of sex distribution, age, educational level, and scores on the Hamilton Anxiety Scale (HAMA) and Hamilton Depression Scale (HAMD), with no statistically significant differences observed (all *p* > 0.05). However, significant differences (*p* < 0.01) were observed between the groups in terms of cognitive function and daily living abilities. Specifically, the AD group performed significantly worse than the HC group on the Mini-Mental State Examination (MMSE), Montreal Cognitive Assessment (MoCA), Auditory Verbal Learning Test-Huashan version short-term delayed recall (AVLT-H-SR) and long-term delayed recall (AVLT-H-LR), Boston Naming Test (BNT), Trail Making Test Part B (TMT-B) completion time, Clock Drawing Test (CDT), Clinical Dementia Rating Scale-Sum of Boxes (CDR-SB), and the Alzheimer’s Disease Cooperative Study - Activities of Daily Living scale (ADCS-ADL).

**Table 1:** Characteristics of participants.

Variables	AD (*n* = 42)	HC (*n* = 49)	*F* (*χ*^2^)	*p*
Sex (male), *n* (%)	21(50.0)	27 (55.1)	0.236	0.63
Age, years, Mean ± SD	74.14 ± 5.44	72.67 ± 6.82	1.26	0.27
Education level, Mean ± SD	7.00 ± 2.26	6.61 ± 2.34	0.64	0.42
MMSE, scores, Mean ± SD	17.55 ± 2.46	27.51 ± 1.23	623.03	<0.01
MoCA, scores, Mean ± SD	14.05 ± 2.55	26.65 ± 0.88	1055.50	<0.01
AVLT-H-SR, scores, Mean ± SD	6.02 ± 0.78	8.82 ± 1.51	116.85	<0.01
AVLT-H-LR, scores, Mean ± SD	4.12 ± 0.86	6.94 ± 1.33	138.89	<0.01
BNT, scores, Mean ± SD	21. 29 ± 5.55	28.94 ± 1.18	88.81	<0.01
TMT-B time, seconds, Mean ± SD	239.12 ± 47.28	70.84 ± 34.42	383.82	<0.01
CDT, scores, Mean ± SD	1.64 ± 0.69	3.79 ± 0.41	330.26	<0.01
CDR-SB, scores, Mean ± SD	2.85 ± 1.50	0.20 ± 0.25	147.48	<0.01
HAMD, scores, Mean ± SD	2.95 ± 1.45	2.12 ± 1.725	2.18	0.14
HAMA, scores, Mean ± SD	3.31 ± 1.63	3.08 ± 1.43	0.51	0.48
ADCS-ADL, scores, Mean ± SD	5.24 ± 2.61	0.59 ± 0.64	145.10	<0.01

AD, Alzheimer’s disease; HC, healthy controls; SD, standard deviation; MMSE, Mini-Mental State Examination; MOCA, Montreal Cognitive Assessment; AVLT-H-SR, short-term delayed recall of auditory verbal learning test-Huashan version; AVLT-H-LR, long-term delayed recall of auditory verbal learning test-Huashan version; TMT-B, Trail Making Test-B; BNT, Boston naming test; CDT, clock drawing test; CDR-SB, Clinical Dementia Rating Scale-Sum of Boxes; HAMD, Hamilton Depression Scale; HAMA, Hamilton Anxiety Scale; ADCS-ADL, Alzheimer’s Disease Collaborative Study - Daily Living Ability Scale.

### Surface-based morphometry (SBM) analysis: cortical thickness (CT)

**Figure 1:**
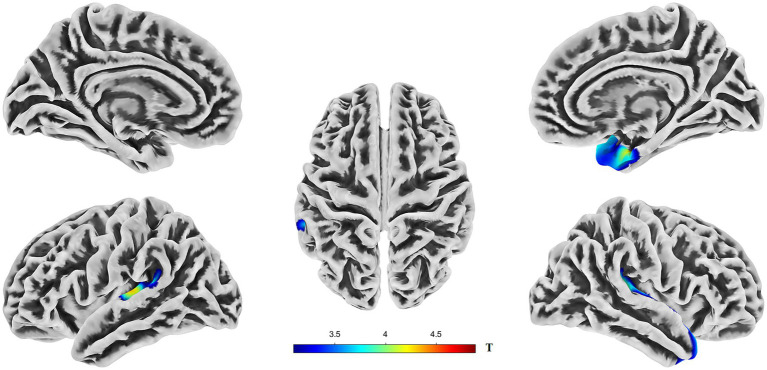
Brain regions showing significant differences in cortical thickness (CT) between AD and HC groups. Colors overlaid on the pial surface rendering represent brain areas where the AD group exhibited significantly reduced CT compared to the HC group (*p* < 0.05, TFCE corrected). The color bars indicate the T-statistic values for this comparison. CT, cortical thickness; TFCE, threshold-free cluster enhancement.

Compared to the HC group, the AD group exhibited significantly reduced cortical thickness (CT). In the left hemisphere, this reduction was primarily localized to Cluster 1 (peak MNI coordinates: −46, −26, and 7), which encompasses the transverse temporal gyrus, superior temporal gyrus, supramarginal gyrus, and insula. The mean CT within Cluster 1 was 2.23 ± 0.14 mm in the AD group and 2.39 ± 0.15 mm in the HC group. In the right hemisphere, significantly reduced CT in the AD group was predominantly observed in Cluster 2 (peak MNI coordinates: 41, −22, and 6), involving the superior temporal gyrus, temporal pole, entorhinal cortex, transverse temporal gyrus, insula, supramarginal gyrus, and fusiform gyrus. The mean CT within Cluster 2 was 2.77 ± 0.23 mm for the AD group and 3.01 ± 0.17 mm for the HC group (see Figure 1, Supplementary Figure S2, and Table 2). Box plots corroborate the SBM findings, demonstrating a marked reduction in mean cortical thickness in the AD group compared to the HC group across the identified regions in both hemispheres (see Supplementary Figure S1 for details).

**Table 2:** Brain regions with reduced cortical thickness in the AD-HC groups.

Cluster	Hemisphere	*T*-value	Size	Peak MNI coordinate	Mean CT in AD group (mm)	Mean CT in HC group (mm)	Overlap of atlas	Region
Cluster 1	L	4.6	472	−46, −26, 7	2.23 ± 0.14	2.39 ± 0.15		
							38%	Left transverse temporal
							49%	Left superior temporal
							12%	Left supramarginal
							1%	Left insula
Cluster 2	R	5.0	1,365	41, −22, 6	2.77 ± 0.23	3.01 ± 0.17		
		

			39%	Right superior temporal
		

			12%	Right temporal pole
		

	8%	Right entorhinal
		

	10%	Right transverse temporal
		

	14%	Right insula
		

	9%	Right supramarginal
		

	5%	Right fusiform

AD, Alzheimer’s disease; HC, Health control; CT, Cortical thickness.

**Figure 2:**
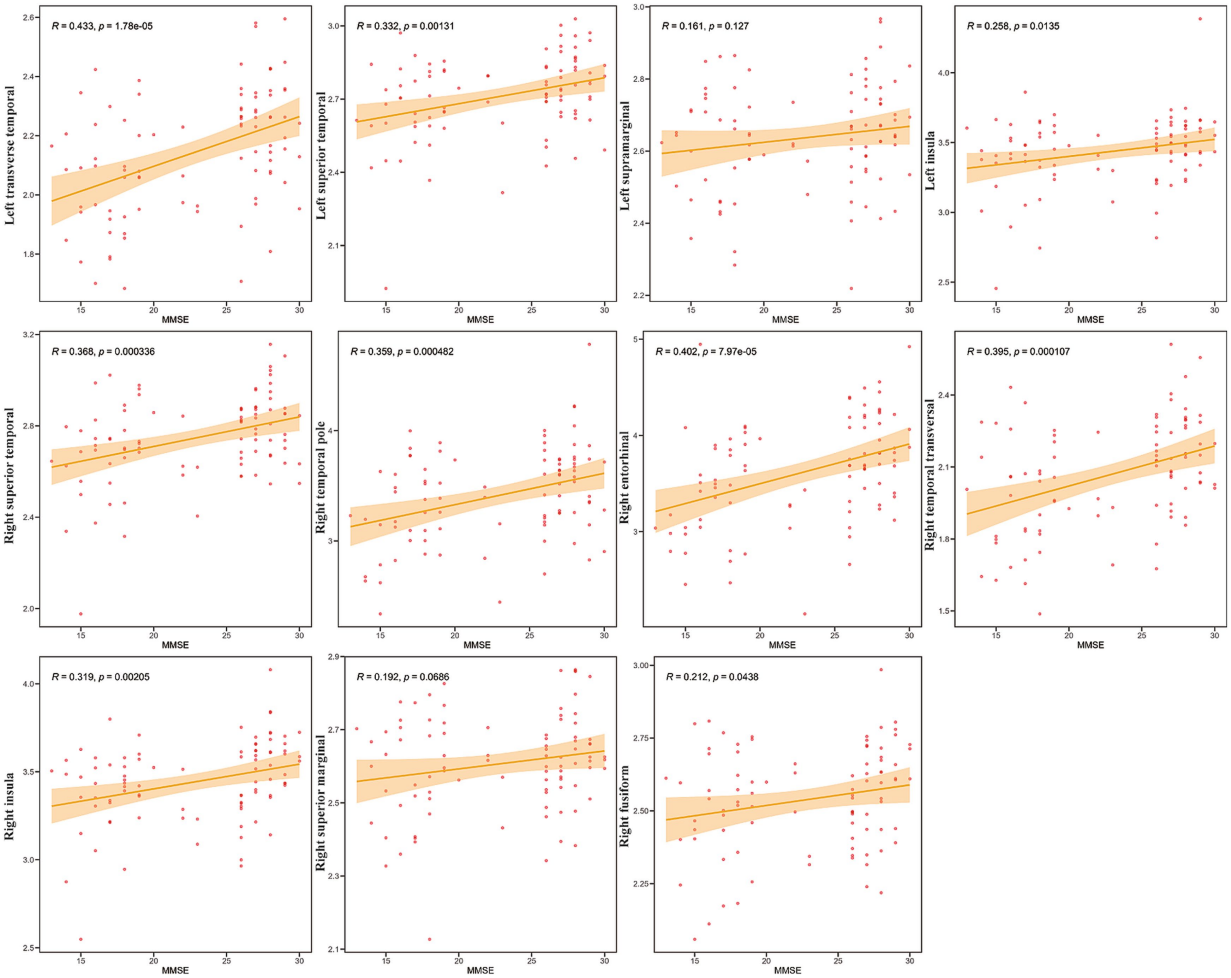
Correlation between mini-mental state examination (MMSE) scores and CT in specific Regions of Interest (ROIs). Scatter plots showing the relationship between cortical thickness (Y-axis) and MMSE scores (X-axis) for the 11 selected ROIs (labeled on the Y-axis). Each red dot represents an individual participant (*N* = 42). The solid orange line indicates the linear regression fit, and the shaded area represents the 95% confidence interval. Pearson’s correlation coefficients (*R*) and the corresponding *p*-values (*p*) are displayed in each subplot.

To investigate the relationship between cognitive function and specific brain metrics, we performed correlation analyses between Mini-Mental State Examination (MMSE) scores and CT in 11 selected Regions of Interest (ROIs) (Figure 2). Significant positive correlations were observed between MMSE scores and CT in multiple brain regions. The strongest associations were found in the left transverse temporal gyrus (*R* = 0.433, *p* = 1.78e-05), the right entorhinal cortex (*R* = 0.402, *p* = 7.97e-05), and the right transverse temporal gyrus (*R* = 0.395, *p* = 0.000107). In contrast, the correlations between MMSE scores and CT in the left supramarginal gyrus (R = 0.161, *p* = 0.127) and the right supramarginal gyrus (R = 0.192, *p* = 0.069) did not reach statistical significance (*p* > 0.05).

These findings indicate that, in this sample, higher cognitive performance, as measured by the MMSE, is associated with greater CT primarily in the temporal and insular regions, while no significant association was found in the supramarginal gyri. Notably, correlations between MoCA scores and these brain regions showed a similar pattern to those observed with the MMSE (Supplementary Figure S4).

### Voxel-based morphometry (VBM) analysis: gray matter volume (GMV)

**Figure 3:**
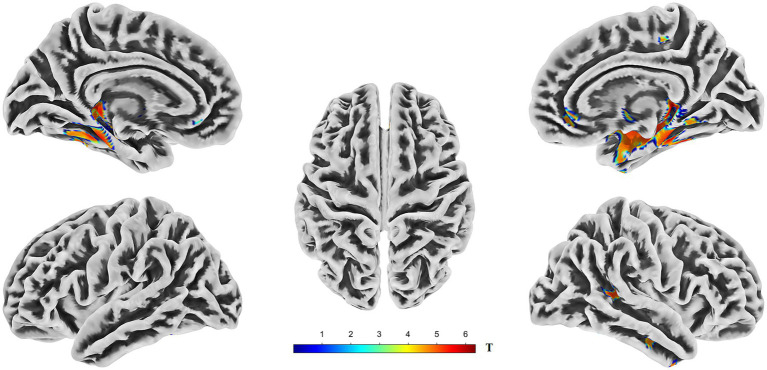
Brain regions exhibiting significantly lower gray matter volume (GMV) in AD compared to HC. The figure displays the results of a voxel-wise comparison of GMV between the two groups rendered onto five views of a template brain surface. The color overlay indicates clusters in which AD showed significantly reduced GMV relative to HC. The color bar represents the *T*-statistic values for these significant differences, with warmer colors (yellow to red) indicating a stronger statistical significance. Statistical significance was determined using a threshold of *p* < 0.05, corrected for multiple comparisons across the whole brain using the Threshold-Free Cluster Enhancement (TFCE) method. GMV, gray matter volume; TFCE, threshold-free cluster enhancement.

VBM analysis revealed significant gray matter volume (GMV) reductions in the AD group compared to those in the HC group. In the left hemisphere, these reductions were primarily observed in the hippocampus, fusiform gyrus, parahippocampal gyrus, posterior temporal lobe, inferior temporal gyrus, and amygdala. In the right hemisphere, significant GMV reductions were predominantly observed in the hippocampus, parahippocampal gyrus, fusiform gyrus, limbic lobe regions, posterior temporal lobe, middle temporal gyrus, inferior temporal gyrus, corticohypothalamus, and amygdala (Figure 3, Supplementary Figures S2, S3, and Table 3).

**Table 3:** Brain regions with GMV loss in the AD-HC groups.

Cluster	*T*-value	Size	Peak MNI coordinate	Overlap of atlas	Region
Cluster 1	7.2	4,221	21, −33, 2		
				16%	Right hippocampus
				8%	Right parahippocampal
				12%	Right fusiform
				17%	Right posterior temporal lobe
				9%	Right amygdala
				6%	Right thalamus
				11%	Right inferior temporal
				7%	Right limbic lobe
				5%	Middle temporal gyrus
Cluster 2	6.5	2,231	−46.5, −53, −27		
				7%	Left hippocampus
				7%	Left parahippocampal
				16%	Left fusiform
				8%	Left amygdala
				37%	Left posterior temporal lobe
				8%	Left inferior temporal
				9%	Left thalamus

GMV, Gray matter volume; AD, Alzheimer’s disease; HC, Health control; MNI: Montreal Neurological Institute.

**Figure 4:**
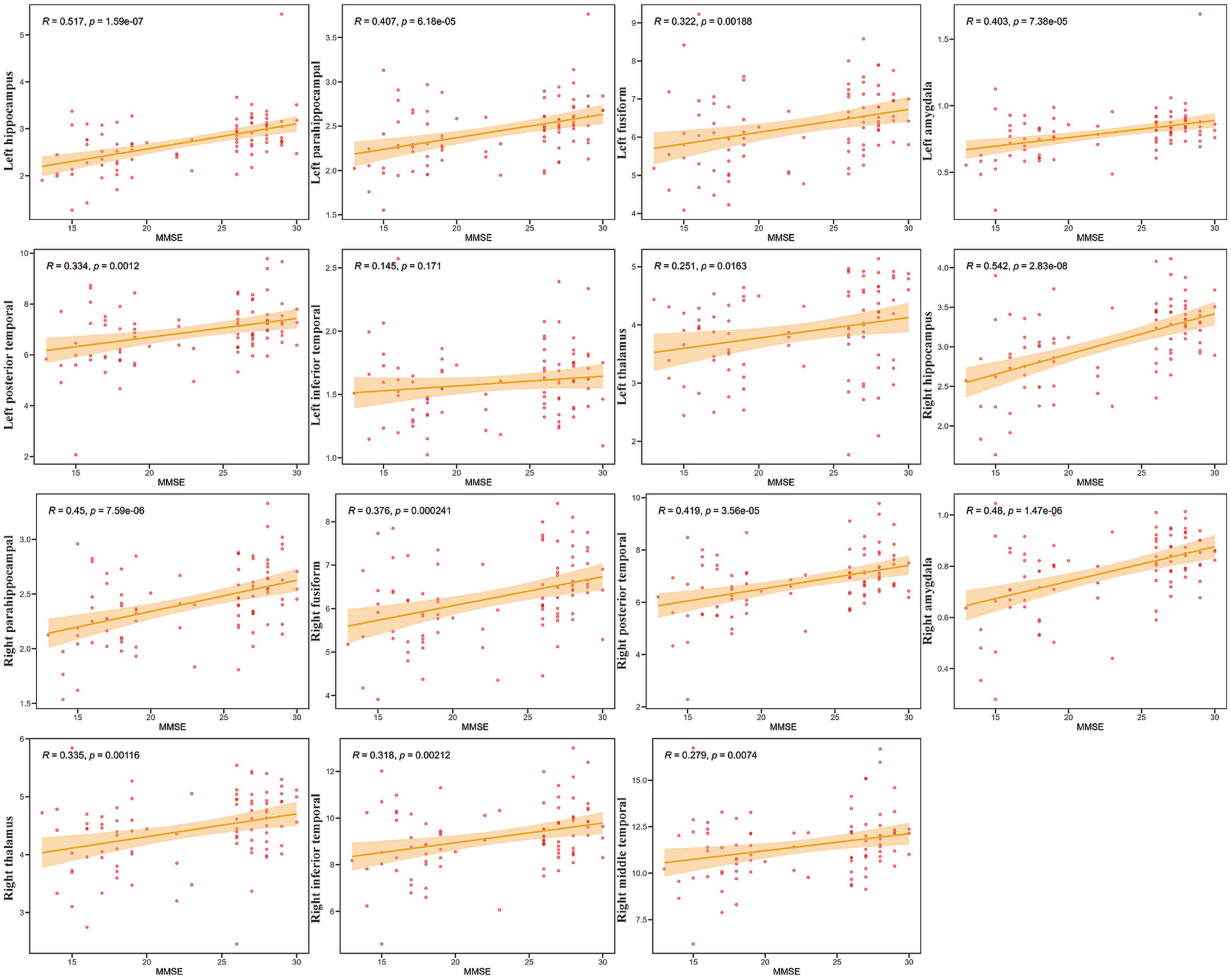
Correlations between mini-mental state examination (MMSE) scores and GMV in specific Regions of Interest (ROIs). Scatter plots showing the relationship between gray matter volume (*Y*-axis) and MMSE scores (*X*-axis) for 15 selected ROIs (labeled on the *Y*-axis). Each red dot represents an individual participant (*N* = 42). The solid orange line indicates the linear regression fit, and the shaded area represents the 95% confidence interval. Pearson’s correlation coefficients (*R*) and the corresponding *p*-values (*p*) are displayed in each subplot.

Further analyses explored the relationship between cognitive performance (using MMSE scores) and GMV across an additional set of 15 ROIs, focusing on the medial temporal lobe structures, the thalamus, and other temporal regions (Figure 4).

Strong and highly significant positive correlations were observed bilaterally between MMSE scores and GMV in the hippocampus (Right: *R* = 0.542, *p* = 2.83e-08; Left: *R* = 0.517, *p* = 1.59e-07), amygdala (Right: *R* = 0.48, *p* = 1.47e-06; Left: *R* = 0.403, *p* = 7.38e-05), and parahippocampal gyrus (Right: *R* = 0.45, *p* = 7.59e-06; Left: *R* = 0.407, *p* = 6.18e-05). The correlation for the left inferior temporal gyrus, however, was not statistically significant (*R* = 0.145, *p* = 0.171). Collectively, these results demonstrate widespread positive relationships between MMSE scores and GMV in critical memory-associated structures (hippocampus, amygdala, parahippocampal gyrus), as well as other temporal and subcortical (thalamus) regions examined. Furthermore, the correlation between MoCA scores and GMV within the ROIs exhibited similar characteristics (Supplementary Figure S5).

The results from both analytical approaches consistently indicated that the temporal lobe is the main region of AD-related atrophy.

## Discussion

### Regional brain atrophy patterns identified by SBM and VBM

Our analysis revealed that consistent with the progression of AD, CT decreased predominantly in the transverse temporal gyrus, superior temporal gyrus, superior marginal gyrus, insula, temporal pole, entorhinal cortex, and fusiform gyrus. These findings align with those of previous studies that employed SBM to investigate brain atrophy patterns in patients with AD (Williams et al., 2021; Sattari et al., 2022; Williams et al., 2023). Similarly, reductions in GMV were primarily observed in the hippocampus, parahippocampal gyrus, posterior temporal lobe, inferior temporal gyrus, middle temporal gyrus, limbic lobe, fusiform gyrus, subcortical amygdala, and thalamus. This pattern is consistent with prior research utilizing Voxel-Based Morphometry (VBM) analysis (Knudsen et al., 2022; Zhu et al., 2019; Xiong et al., 2023).

Regions exhibiting GMV atrophy (identified via VBM) and reduced CT (identified via SBM) largely overlapped and were predominantly located in the temporal, medial temporal, and parietal lobes, which are known to be closely associated with cognitive function. Although SBM analysis primarily quantifies CT, it is less effective for measuring subcortical GMV. In contrast, VBM analysis allows for the assessment of both cortical and subcortical GMV (Pergher et al., 2019). The findings from both analyses demonstrated substantial consistency in cortical atrophy patterns, corroborating the results reported by Yan et al. (2024) and Goto et al. (2022).

### Correlation between brain atrophy measures and cognitive scores

We performed correlation analyses between the identified regions of CT and GMV reduction and scores on the Mini-Mental State Examination (MMSE) and Montreal Cognitive Assessment (MoCA) (Table 4). Significant positive correlations (indicating that greater atrophy corresponds to lower scores) with MMSE scores were observed primarily in the right entorhinal cortex, right transverse temporal gyrus, bilateral superior temporal gyrus, bilateral amygdala, bilateral hippocampus, bilateral fusiform gyrus, right inferior temporal gyrus, right middle temporal gyrus, bilateral parahippocampal gyrus, and bilateral thalamus (*p* < 0.05). Similarly, regions that correlated significantly with MoCA scores included the right entorhinal cortex, right transverse temporal gyrus, right insula, bilateral superior temporal gyrus, bilateral amygdala, bilateral hippocampus, bilateral fusiform gyrus, bilateral inferior temporal gyrus, right middle temporal gyrus, bilateral parahippocampal gyrus, and bilateral thalamus (*p* < 0.05). Overall, the distribution of cortical regions and subcortical gray matter nuclei showing correlations with MMSE and MoCA scores was largely similar. These findings align with previous research by Sun et al. (2019) and Huang et al. (2020), who also reported correlations between cognitive performance and atrophy in specific brain regions (Huang et al., 2020; Sun et al., 2019).

**Table 4:** Correlation analysis of CT and GMV reduction in brain regions and MMSE and MoCA scores.

Region	MoCA		MMSE	
	*r*	*p*	*r*	*p*
Right entorhinal	0.361	0.000	0.402	0.000
Right temporal pole	0.327	0.002	0.359	0.000
Right transverse temporal	0.370	0.000	0.395	0.000
Left insula	0.240	0.022	0.258	0.014
Right insula	0.282	0.007	0.319	0.002
Left superior temporal	0.304	0.003	0.332	0.001
Right superior temporal	0.340	0.001	0.368	0.000
Left supramarginal	0.118	0.267	0.161	0.127
Right supramarginal	0.153	0.149	0.192	0.069
Left amygdala	0.389	0.000	0.403	0.000
Right amygdala	0.468	0.000	0.480	0.000
Left hippocampus	0.517	0.000	0.517	0.000
Right hippocampus	0.554	0.000	0.542	0.000
Left fusiform	0.319	0.002	0.322	0.002
Right fusiform	0.368	0.000	0.376	0.000
Left inferior temporal	0.158	0.002	0.145	0.171
Right inferior temporal	0.318	0.002	0.318	0.002
Right middle temporal gyrus	0.248	0.018	0.279	0.007
Left parahippocampal	0.390	0.000	0.407	0.000
Right parahippocampal	0.422	0.000	0.450	0.000
Left posterior temporal lobe	0.317	0.002	0.334	0.001
Right posterior temporal lobe	0.391	0.000	0.419	0.000
Right thalamus	0.330	0.001	0.335	0.001
Left thalamus	0.238	0.023	0.251	0.016

CT, Cortical Thickness; GMV, Gray Matter Volume; MMSE, Mini-Mental State Examination; MoCA, Montreal Cognitive Assessment.

### Diagnostic utility assessed by ROC analysis

**Figure 5:**
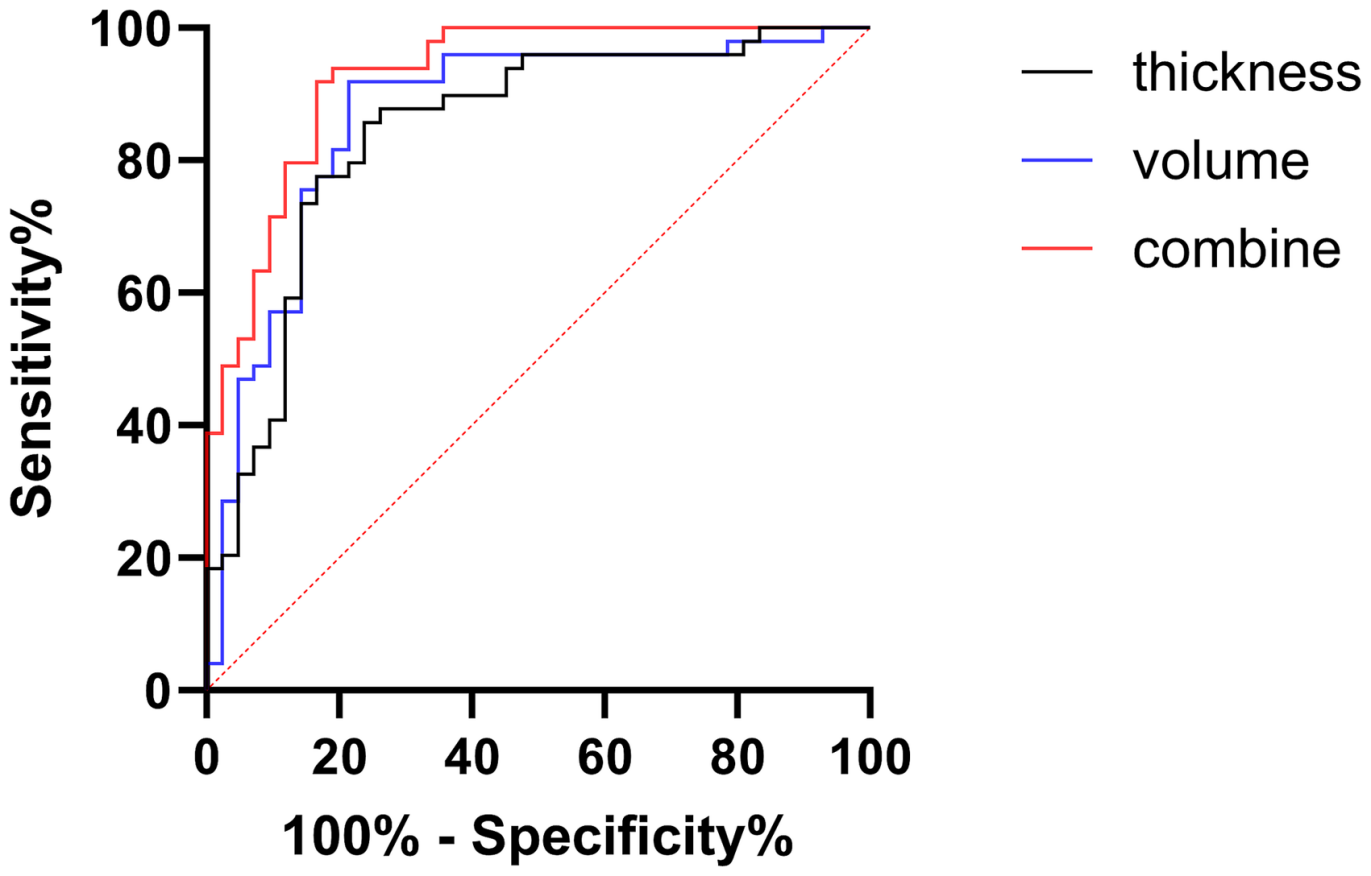
Receiver operating characteristic (ROC) analysis for diagnosing cognitive impairment. Curves illustrate the diagnostic performance based on CT (black line), GMV (blue line), and a combined model (CT + GMV, red line). Sensitivity (%) is plotted against 100% specificity (%). The diagonal dashed line represents the chance level (AUC = 0.5). Higher performance is indicated by the curves closer to the top left. CT, cortical thickness; GMV, gray matter volume.

To evaluate the diagnostic potential of these neuroimaging markers, we established predictive models for cognitive dysfunction based on CT, GMV, and their combination, generating corresponding Receiver Operating Characteristic (ROC) curves (Figure 5). The Area Under the Curve (AUC) values were 0.845 (95% CI: 0.76–0.93, *p* < 0.001) for CT, 0.867 (95% CI: 0.79–0.95, *p* < 0.001) for GMV, and 0.926 (95% CI: 0.87–0.98, *p* < 0.001) for the combined model. The AUC values for both CT (derived from SBM) and GMV (derived from VBM) were substantial and comparable, suggesting that each measure independently holds a significant diagnostic value for cognitive impairment. Notably, the combined CT and GMV model yielded a significantly higher AUC than either measure alone (*p* < 0.05), indicating a superior diagnostic performance for predicting cognitive impairment. This finding is consistent with those of previous studies by Ma et al. (2020) and Chen et al. (2021), supporting the notion that integrating cortical thickness and gray matter volume provides a more robust neuroimaging biomarker for AD-related cognitive decline.

While our findings corroborate the seminal work of Dickerson et al. (2009) on AD-related cortical thinning patterns, we extend these observations by demonstrating convergent validity between the SBM and VBM modalities. In particular, our dual-modality approach resolves the methodological discrepancy reported by Fjell et al. (2014) regarding the subcortical measurement sensitivity. The striking overlap in temporal lobe atrophy between modalities (AUC > 0.85 for both) provides stronger evidence than single-modality studies, such as those by Willette et al. (2014), supporting the temporal lobe as the most vulnerable epicenter in early AD progression.

These findings have immediate clinical relevance; the integrated model could enhance early diagnosis in ambiguous cases by detecting subtle structural changes missed by conventional MRI assessment, particularly for patients with mild cognitive impairment (MCI) who may benefit from closer monitoring or early interventions. For broader clinical adoption, future efforts should focus on standardizing automated analysis pipelines and establishing age-adjusted normative databases, which are critical steps toward implementing this approach as a cost-effective tool for risk stratification and therapeutic monitoring in memory clinics.

Although this study provides important insights into AD-related brain atrophy patterns, several limitations should be considered. The small sample size may have limited our ability to detect subtle regional atrophy patterns, particularly in smaller subcortical structures. Additionally, the absence of fluid biomarker data, such as CSF or plasma Aβ and tau levels, prevents a direct correlation between structural changes and underlying AD pathology. Finally, individual variability in neuroanatomy, especially in highly variable regions, such as the temporal pole, could introduce measurement variability that warrants further investigation in future studies using advanced registration techniques.

## Conclusion

In patients with AD, VBM and SBM analyses revealed overlapping patterns of cortical GMV and CT reductions, respectively. However, VBM offers the additional advantage of assessing atrophy in the subcortical gray matter structures implicated in cognition. Correlation analyses confirmed that reduced volume and thickness in these identified regions were positively associated with the MMSE and MoCA scores, underscoring their functional relevance. Furthermore, ROC analysis demonstrated that, while both CT and GMV individually possess diagnostic value, a combined model integrating both measures significantly enhances the prediction of cognitive impairment. This integrated approach may therefore offer improved utility for clinical assessment and future research into AD diagnostics, potentially leading to more accurate and earlier identification of individuals at risk.

## Data Availability

The raw data supporting the conclusions of this article will be made available by the authors, without undue reservation.

## References

[ref1] Braak H. Braak E. (1991). Neuropathological stageing of Alzheimer-related changes. Acta Neuropathol. 82, 239–259. doi: 10.1007/BF00308809, PMID: 1759558

[ref2] Chen W. Li S. Ma Y. Lv S. Wu F. Du J.. (2021). A simple nomogram prediction model to identify relatively young patients with mild cognitive impairment who may progress to Alzheimer's disease. J. Clin. Neurosci. 91, 62–68. doi: 10.1016/j.jocn.2021.06.026, PMID: 34373060

[ref3] Chen Z. Xie Q. Wang J. Wang Y. Zhang H. Li C.. (2024). Mapping grey matter and cortical thickness alterations associated with subjective cognitive decline and mild cognitive impairment among rural-dwelling older adults in China: a population-based study. Neuroimage Clin. 44:103691. doi: 10.1016/j.nicl.2024.103691, PMID: 39488196 PMC11566878

[ref4] Chrzan R. Gleń A. Bryll A. Urbanik A. (2019). Computed tomography assessment of brain atrophy in centenarians. Int. J. Environ. Res. Public Health 16:3659. doi: 10.3390/ijerph16193659, PMID: 31569457 PMC6801833

[ref5] Contador J. Pérez-Millán A. Tort-Merino A. Balasa M. Falgàs N. Olives J.. (2021). Longitudinal brain atrophy and CSF biomarkers in early-onset Alzheimer's disease. Neuroimage Clin. 32:102804. doi: 10.1016/j.nicl.2021.102804, PMID: 34474317 PMC8405839

[ref6] Cooper J. (2001). Diagnostic and statistical manual of mental disorders (4th edn, text revision) (DSM–IV–TR) Washington, DC: American Psychiatric Association 2000. 943 pp. £ 39.99 (hb). 0 89042 025 4. Br. J. Psychiatry 179:85.

[ref7] Cui X. Li M. Lei G. Wang J. Pan J. Zhu S.. (2024). Differences in cerebral structure among patients with amnestic mild cognitive impairment and patients with Alzheimer's disease. Front. Aging Neurosci. 16:1453051. doi: 10.3389/fnagi.2024.1453051, PMID: 39697482 PMC11652504

[ref8] Devanand D. P. Bansal R. Liu J. Hao X. Pradhaban G. Peterson B. S. (2012). MRI hippocampal and entorhinal cortex mapping in predicting conversion to Alzheimer's disease. NeuroImage 60, 1622–1629. doi: 10.1016/j.neuroimage.2012.01.075, PMID: 22289801 PMC3320768

[ref9] Dickerson B. C. Bakkour A. Salat D. H. Feczko E. Pacheco J. Greve D. N.. (2009). The cortical signature of Alzheimer's disease: regionally specific cortical thinning relates to symptom severity in very mild to mild AD dementia and is detectable in asymptomatic amyloid-positive individuals. Cereb. Cortex 19, 497–510. doi: 10.1093/cercor/bhn113, PMID: 18632739 PMC2638813

[ref10] Dubois B. Feldman H. H. Jacova C. Hampel H. Molinuevo J. L. Blennow K.. (2014). Advancing research diagnostic criteria for Alzheimer's disease: the IWG-2 criteria. Lancet Neurol. 13, 614–629. doi: 10.1016/S1474-4422(14)70090-0, PMID: 24849862

[ref11] Fjell A. M. Westlye L. T. Grydeland H. Amlien I. Espeseth T. Reinvang I.. (2014). Accelerating cortical thinning: unique to dementia or universal in aging? Cereb. Cortex 24, 919–934. doi: 10.1093/cercor/bhs379, PMID: 23236213 PMC3948495

[ref12] Goto M. Abe O. Hagiwara A. Fujita S. Kamagata K. Hori M.. (2022). Advantages of using both voxel- and surface-based morphometry in cortical morphology analysis: a review of various applications. Magn. Reson. Med. Sci. 21, 41–57. doi: 10.2463/mrms.rev.2021-0096, PMID: 35185061 PMC9199978

[ref13] Huang L. Chen K. Hu X. Guo Q. (2020). Differential atrophy in the hippocampal subfield volumes in four types of mild dementia. Front. Neurosci. 14:699. doi: 10.3389/fnins.2020.00699, PMID: 32742253 PMC7364129

[ref14] Jack C. R. Albert M. S. Knopman D. S. McKhann G. M. Sperling R. A. Carrillo M. C.. (2011). Introduction to the recommendations from the National Institute on Aging-Alzheimer's Association workgroups on diagnostic guidelines for Alzheimer's disease. Alzheimers Dement. 7, 257–262. doi: 10.1016/j.jalz.2011.03.004, PMID: 21514247 PMC3096735

[ref15] Kim S. W. Kim D. H. Hong J. Y. Mun K. R. Jung D. Hong I.. (2025). Gait impairment associated with neuroimaging biomarkers in Alzheimer's disease. Sci. Rep. 15:5539. doi: 10.1038/s41598-025-90020-4, PMID: 39953283 PMC11828857

[ref16] Knudsen L. V. Gazerani P. Duan Y. Michel T. M. Vafaee M. S. (2022). The role of multimodal MRI in mild cognitive impairment and Alzheimer's disease. J. Neuroimaging 32, 148–157. doi: 10.1111/jon.12940, PMID: 34752671

[ref17] Longhurst J. K. Sreenivasan K. R. Kim J. Cummings J. L. John S. E. Poston B.. (2023). Cortical thickness is related to cognitive-motor automaticity and attention allocation in individuals with Alzheimer's disease: a regions of interest study. Exp. Brain Res. 241, 1489–1499. doi: 10.1007/s00221-023-06618-5, PMID: 37085647

[ref18] Ma Z. Jing B. Li Y. Yan H. Li Z. Ma X.. (2020). Identifying mild cognitive impairment with random Forest by integrating multiple MRI morphological metrics. J. Alzheimers Dis. 73, 991–1002. doi: 10.3233/JAD-190715, PMID: 31884464

[ref19] Pergher V. Demaerel P. Soenen O. Saarela C. Tournoy J. Schoenmakers B.. (2019). Identifying brain changes related to cognitive aging using VBM and visual rating scales. Neuroimage Clin. 22:101697. doi: 10.1016/j.nicl.2019.101697, PMID: 30739844 PMC6370556

[ref20] Ramirez M. K. Phipps C. J. Murman D. L. Beadle J. N. Phatak V. S. Warren D. E. (2025). Structural neuroimaging correlates of neuropsychiatric symptoms in Alzheimer's disease: a systematic literature review. Dement. Geriatr. Cogn. Disord. 1-15, 1–22. doi: 10.1159/000543160, PMID: 39938492 PMC12324975

[ref21] Roalf D. R. Moberg P. J. Xie S. X. Wolk D. A. Moelter S. T. Arnold S. E. (2013). Comparative accuracies of two common screening instruments for classification of Alzheimer's disease, mild cognitive impairment, and healthy aging. Alzheimers Dement. 9, 529–537. doi: 10.1016/j.jalz.2012.10.001, PMID: 23260866 PMC4036230

[ref22] Sakurai R. Bartha R. Montero-Odasso M. (2019). Entorhinal cortex volume is associated with dual-task gait cost among older adults with MCI: results from the gait and brain study. J. Gerontol. A Biol. Sci. Med. Sci. 74, 698–704. doi: 10.1093/gerona/gly084, PMID: 29767690 PMC6477635

[ref23] Salimi-Khorshidi G. Smith S. M. Nichols T. E. (2011). Adjusting the effect of nonstationarity in cluster-based and TFCE inference. NeuroImage 54, 2006–2019. doi: 10.1016/j.neuroimage.2010.09.088, PMID: 20955803

[ref24] Sattari N. Faeghi F. Shekarchi B. Heidari M. H. (2022). Assessing the changes of cortical thickness in Alzheimer disease with MRI using Freesurfer software. Basic Clin. Neurosci. 13, 185–192. doi: 10.32598/bcn.2021.1779.1, PMID: 36425945 PMC9682320

[ref25] Sun P. Lou W. Liu J. Shi L. Li K. Wang D.. (2019). Mapping the patterns of cortical thickness in single- and multiple-domain amnestic mild cognitive impairment patients: a pilot study. Aging (Albany NY) 11, 10000–10015. doi: 10.18632/aging.102362, PMID: 31756169 PMC6914405

[ref26] Willette A. A. Calhoun V. D. Egan J. M. Kapogiannis D. (2014). Prognostic classification of mild cognitive impairment and Alzheimer's disease: MRI independent component analysis. Psychiatry Res. 224, 81–88. doi: 10.1016/j.pscychresns.2014.08.005, PMID: 25194437 PMC4586157

[ref27] Williams M. E. Elman J. A. Bell T. R. Dale A. M. Eyler L. T. Fennema-Notestine C.. (2023). Higher cortical thickness/volume in Alzheimer's-related regions: protective factor or risk factor? Neurobiol. Aging 129, 185–194. doi: 10.1016/j.neurobiolaging.2023.05.004, PMID: 37343448 PMC10676195

[ref28] Williams M. E. Elman J. A. McEvoy L. K. Andreassen O. A. Dale A. M. Eglit G. M. L.. (2021). 12-year prediction of mild cognitive impairment aided by Alzheimer's brain signatures at mean age 56. Brain Commun. 3:fcab167. doi: 10.1093/braincomms/fcab167, PMID: 34396116 PMC8361427

[ref29] Xiong Y. Ye C. Sun R. Chen Y. Zhong X. Zhang J.. (2023). Disrupted balance of gray matter volume and directed functional connectivity in mild cognitive impairment and Alzheimer's disease. Curr. Alzheimer Res. 20, 161–174. doi: 10.2174/1567205020666230602144659, PMID: 37278043 PMC10514512

[ref30] Yan Y. He X. Xu Y. Peng J. Zhao F. Shao Y. (2024). Comparison between morphometry and radiomics: detecting normal brain aging based on grey matter. Front. Aging Neurosci. 16:1366780. doi: 10.3389/fnagi.2024.1366780, PMID: 38685908 PMC11056505

[ref31] Yuan S. Gong Y. Zhang Y. Cao W. Wei L. Sun T.. (2025). Brain structural alterations in young women with premature ovarian insufficiency: implications for dementia risk. Alzheimers Dement. 21:e70111. doi: 10.1002/alz.70111, PMID: 40145307 PMC11947759

[ref32] Zhang B. Lin L. Wu S. (2021). A review of brain atrophy subtypes definition and analysis for Alzheimer's disease heterogeneity studies. J. Alzheimers Dis. 80, 1339–1352. doi: 10.3233/JAD-201274, PMID: 33682711

[ref33] Zhou Y. (2021). Imaging and multiomic biomarker applications: advances in early Alzheimer’s disease. Hauppauge, NY, USA: Nova Science Publishers.

[ref34] Zhu J. Zhu D. M. Zhang C. Wang Y. Yang Y. Yu Y. (2019). Quantitative prediction of individual cognitive flexibility using structural MRI. Brain Imaging Behav. 13, 781–788. doi: 10.1007/s11682-018-9905-1, PMID: 29855990

[ref35] Zhuang K. Chen X. Cassady K. E. Baker S. L. Jagust W. J. (2022). Metacognition, cortical thickness, and tauopathy in aging. Neurobiol. Aging 118, 44–54. doi: 10.1016/j.neurobiolaging.2022.06.007, PMID: 35868093 PMC9979699

