# Supplementary material for: Voxel- and surface-based morphometry in the cortical thickness and cortical and subcortical gray matter volume in patients with mild-to-moderate Alzheimer’s disease
Source: Front Aging Neurosci. 2025 Jun 25;17:1546977. doi: 10.3389/fnagi.2025.1546977 (PMC12238721; doi:10.3389/fnagi.2025.1546977)
Supplement: Supplementary file 1 [file Supplementary_file_1.pdf]

Supplementary Materials

Voxel-based and surface-based morphometry in the cortical thickness, and cortical and subcortical grey matter volume in patients with mild to moderate Alzheimer's disease

Kaidi Li[1,2], Dingling Xie[3], Zhengyong Zhang[4], Chunyu Fu[5,2], Chunyang Li[6, *]

1 Department of Neurology, Affiliated Hospital of Inner Mongolia Medical University, Hohhot 010030, the Nei Monggol Autonomous Region, P.R. China.

2 Inner Mongolia Regional Center for Neurological Disorders, Hohhot 010030, the Nei Monggol Autonomous Region, P.R. China.

3 Department of Neurology, Xianning Central Hospital (The First Affiliated Hospital Of Hubei University Of Science And Technology), Xianning 437100, Hubei, P.R. China.

4 School of Public Health, Xinxiang Medical College, Xinxiang 453003, Henan, P.R. China.

5 Department of Neurosurgery, Affiliated Hospital of Inner Mongolia Medical University, Hohhot 010030, the Nei Monggol Autonomous Region, P.R. China.

6 Department of Neurology, Inner Mongolia Brain Hospital (Third Hospital), Hohhot 010030, the Nei Monggol Autonomous Region, P.R. China.

[*]**Corresponding author:** Chunyang Li, Inner Mongolia Brain Hospital, No.23, Wulanchabu West Road, Xincheng District, Hohhot 010030, the Nei Monggol Autonomous Region, P.R. China.

E-mail: lichunyang770905@163.com

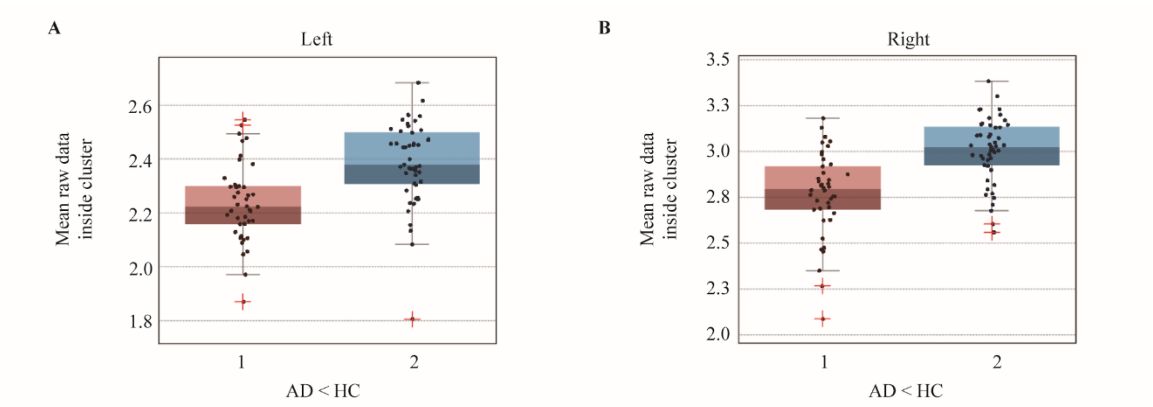

Figure S1. Comparison of mean cortical thickness between AD patients and HC within brain regions showing significant atrophy (AD < HC) identified by Surface-Based Morphometry (SBM). Box plots display the distribution of average cortical thickness within these clusters for

AD (1, red) and HC (2, blue) groups in the **(A)** Left and **(B)** Right hemispheres. Dots represent individual subjects; boxes show median and interquartile range (IQR); crosses denote outliers. AD: Alzheimer's disease; HC: healthy controls.

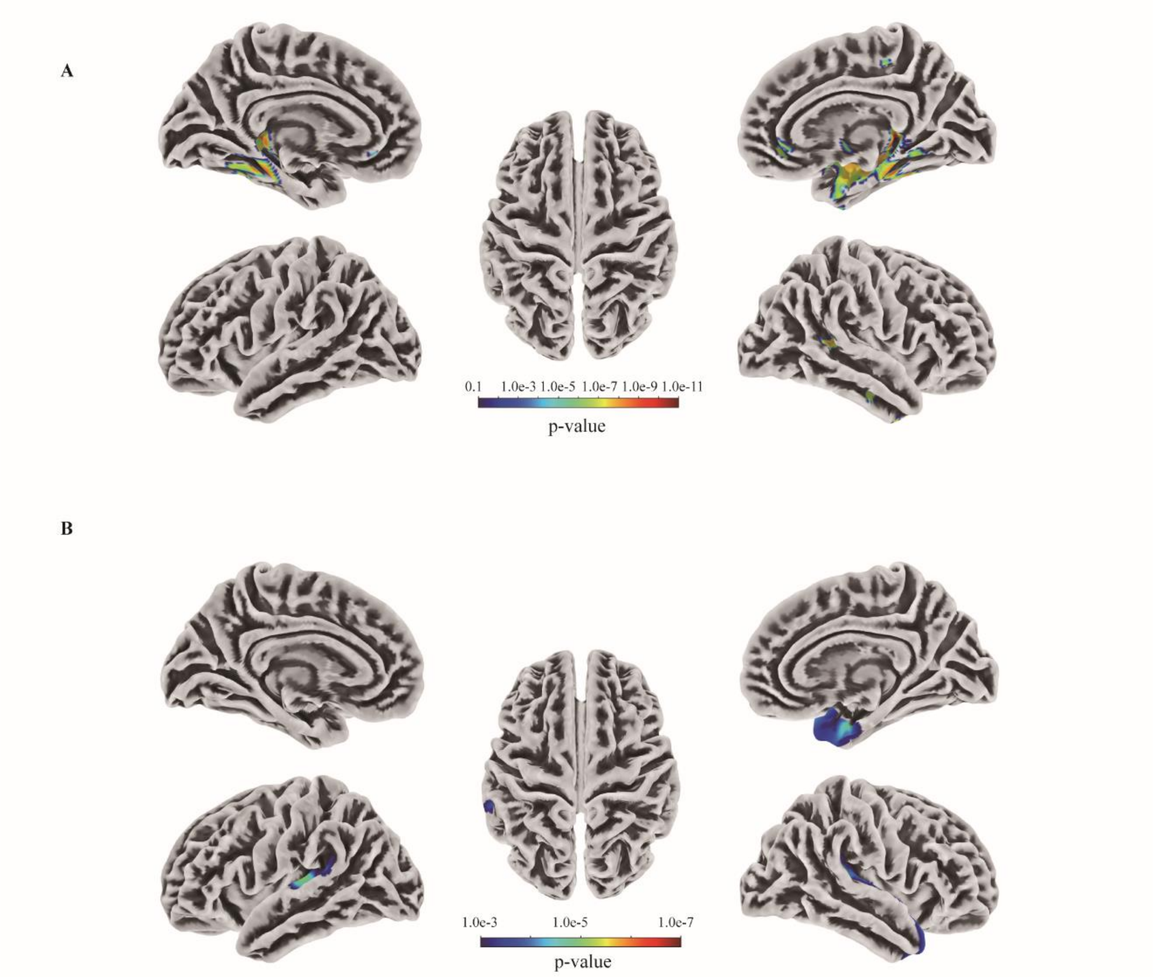

Figure S2. Comparison of brain atrophy patterns between AD patients and HC. **(A)** Voxel-Based Morphometry (VBM) analysis reveals significant gray matter atrophy (p-values indicated by color bar) in AD patients, primarily affecting the temporal lobe. **(B)** Surface-Based Morphometry (SBM) analysis confirms significant cortical atrophy (p-values indicated by color bar) in similar temporal regions in the AD group. Warmer colors denote lower p-values (greater significance). Maps are shown in multiple anatomical views. AD: Alzheimer's disease; HC: healthy controls.

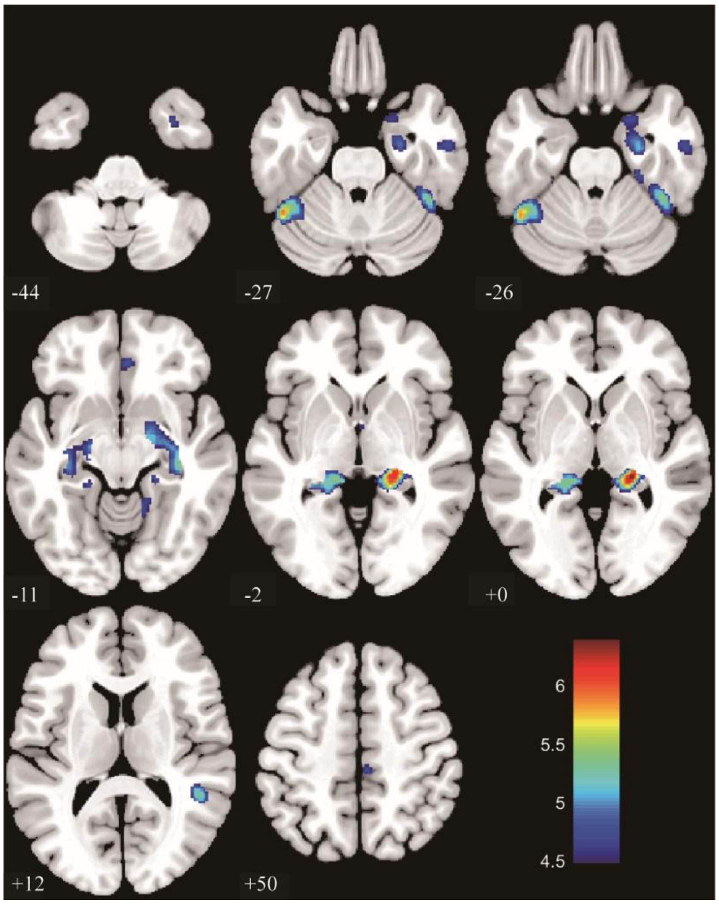

Figure S3. Significant gray matter atrophy in AD vs. HC (VBM analysis). The figure displays a T-map highlighting regions of significantly reduced gray matter volume ($p < 0.05$, FWE-corrected) in the AD group compared to HC, based on Voxel-Based Morphometry (VBM) analysis. These statistical results are overlaid on representative axial brain slices. The axial slices were strategically selected at specific MNI space to best illustrate the spatial extent of the significant findings. The specific coordinates for slices shown are: -44, -27, -26, -11, -2, 0, +12, +50. The color bar indicates the T-values, with warmer colors representing greater statistical significance. AD: Alzheimer's disease; HC: healthy controls.

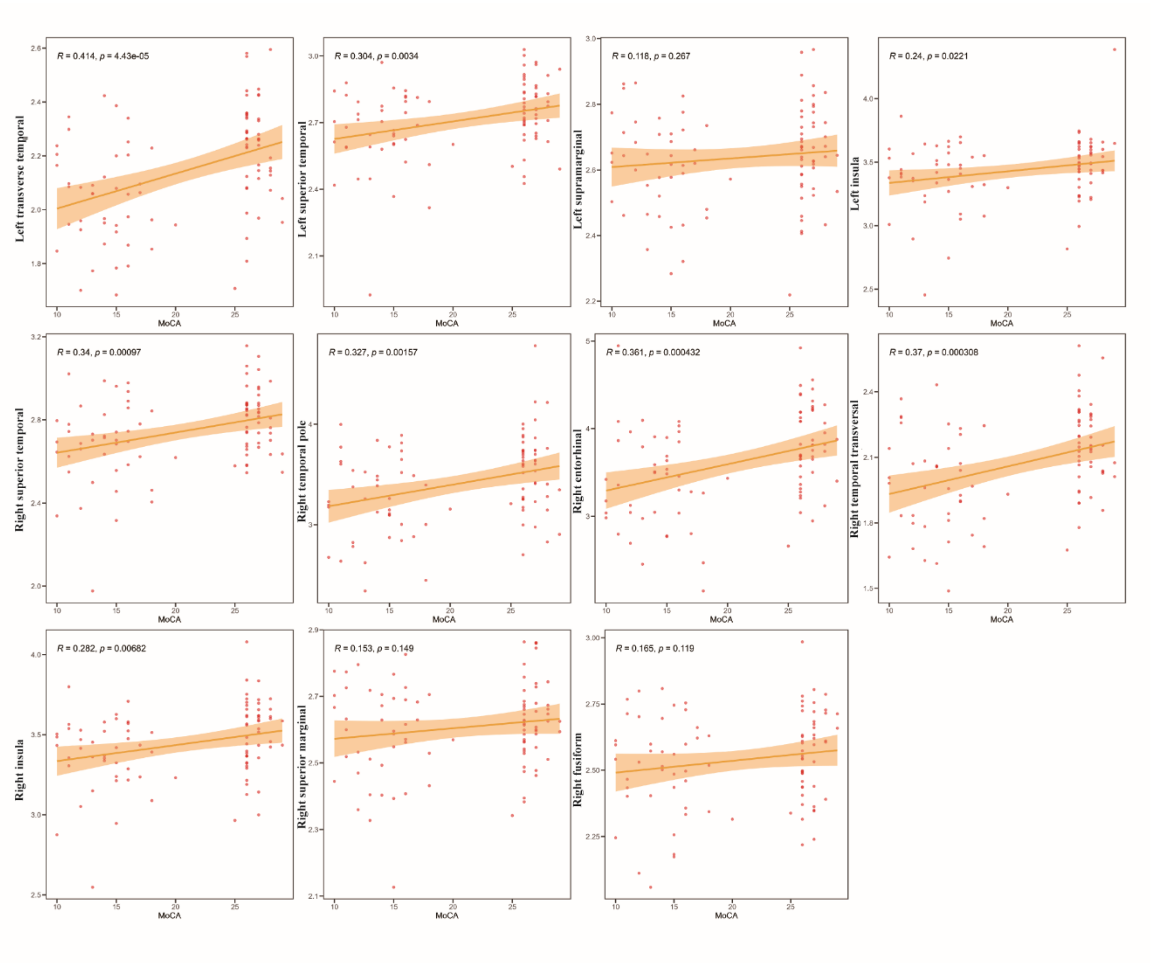

Figure S4. Correlations between Montreal Cognitive Assessment (MoCA) scores and CT in specific Regions of Interest (ROIs). Scatter plots showing the relationship between cortical thickness (Y-axis) and MMSE scores (X-axis) for 11 selected ROIs (labeled on the Y-axis). Each red dot represents an individual participant (N = 42). The solid orange line indicates the linear regression fit, with the shaded area representing the 95% confidence interval. Pearson correlation coefficient (R) and corresponding p-value (p) are displayed within each subplot.

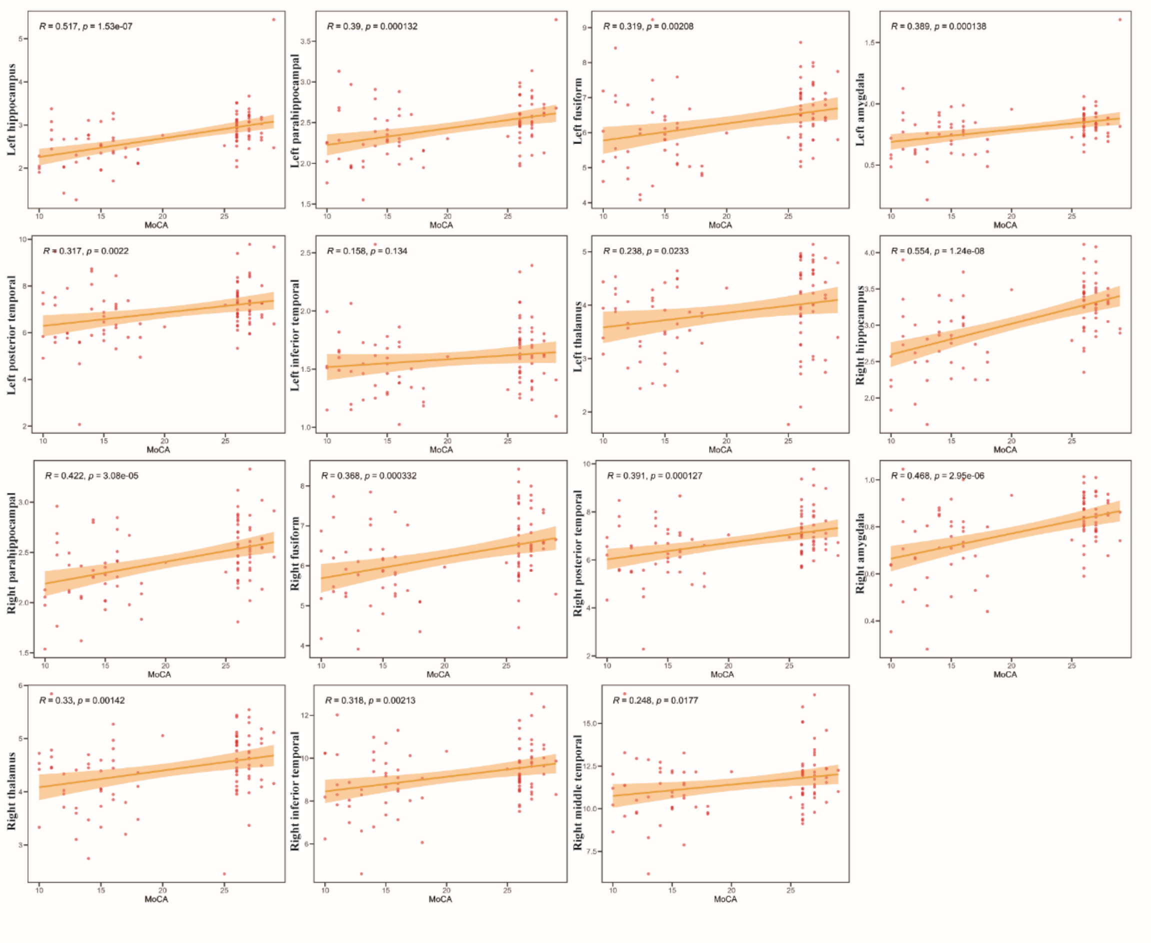

Figure S5. Correlations between Montreal Cognitive Assessment (MoCA) scores and GMV in specific Regions of Interest (ROIs). Scatter plots showing the relationship between grey matter volume (Y-axis) and MMSE scores (X-axis) for 15 selected ROIs (labeled on the Y-axis). Each red dot represents an individual participant (N = 42). The solid orange line indicates the linear regression fit, with the shaded area representing the 95% confidence interval. Pearson correlation coefficient (R) and corresponding p-value (p) are displayed within each subplot.
